# Looseness of the Teeth

**Published:** 1842-06

**Authors:** 


### LOOSENESS OF THE TEETH.

Among the various causes which produce looseness of one or several teeth, none is more common than inflammation of the alveolar processes and sockets. Sometimes this originates in disease of the tooth itself, or of the gums; but in other instances, the diseased process commences in the alveolar periosteum, and by spreading to the sockets and gums, it gives rise to great pain, swelling, and sponginess of the latter, while it eventually detaches the fangs of the teeth implicated in the attack, from the grasp of the sockets, and thus at last the teeth fall out, though in themselves they exhibit no appearance of decay.

The progress of the disease is accompanied by extreme pain, and as a puriform discharge oozes out from between the gums and the inflamed periosteum, many limit their attempts to local means, and often succeed in effecting a cure by frequent applications of leeches to the inflamed gum, and in very obstinate cases, by incisions freely made through the gums and inflamed periosteum. Last year a patient of mine was thus affected, and thus treated, and although under the care of a most skilful surgeon, and eminent as a dentist, he lost successively a left bicuspis and molar of the upper jaw. His sufferings were for a short time relieved by the extraction of each tooth, but in a few days became as agonizing as ever, when finding all the neighbouring teeth loose, and being told that they also must soon be drawn, he had recourse, in despair, to a celebrated homœopathic doctor, whose infinitesimal doses completely failed; for the patient's sufferings were produced by a direct physical cause, which lay far beyond the limits to which the influence of even the most powerful imagination can possibly extend. Happening to mention his wretched state to me, I immediately recollected, that a year before I had successfully treated him for a periostic affection of the sternum and ribs, and that hydriodate of potash was the medicine which served him most. I recommended him to use ten grains of it three times a day, and had the satisfaction of perceiving a daily improvement, so that pain and inflammation soon ceased, and in about ten days the teeth were all fastened.

The periostitis to which this gentleman was liable was of a rheumatic nature; otherwise his constitution was sound, and he was only thirty-four years old.—*Dr. Graves, in Dublin Journal. From the Bulletin of Medical Science.*

